# MLGA-CDRF-YOLO: A Lightweight Target Detection Method of Multi-Scale Group Attention and Channel Dynamic Residual Fusion for Multipole Magnet Collimation

**DOI:** 10.3390/s26175325

**Published:** 2026-08-22

**Authors:** Xiaohui Zhai, Hongbing Xin, Xiaolong Wang, Lingling Men

**Affiliations:** 1 School of Computer and Artificial Intelligence, Beijing Technology and Business University, Beijing 100048, China; zhaixh@st.btbu.edu.cn; 2 Institute of High Energy Physics, Chinese Academy of Sciences, Beijing 100043, China; menll@ihep.ac.cn

**Keywords:** object detection, lightweight neural network, channel attention, multi-scale feature fusion, dynamic residual connection, magnet collimation

## Abstract

A lightweight multi-scale group attention and channel dynamic residual fusion method is proposed to address the challenge of detecting extremely small encoded targets in particle accelerator multipole magnet collimation. In this scenario, the encoded targets occupy merely 0.003% to 0.009% of the total image pixels, rendering conventional visual techniques inadequate for capturing such fine-grained features. The paper proposes MLGA-CDRF-YOLO, an improved detection framework based on YOLOv11s that integrates a lightweight multi-scale group attention mechanism with channel-wise dynamic residual fusion. Specifically, the C2f-CDRF module combines lightweight channel attention with a dynamic residual structure, enhancing feature representation while reducing parameter count and computational complexity. The Multi-scale Lightweight Group Attention (MLGA) module splits input features into groups processed in parallel through a channel attention branch and a multi-scale spatial attention branch, with ChannelShuffle enabling cross-group information exchange, to improve the model’s feature representation capability and generalization performance for complex scenes. Experimental results on the encoded target dataset demonstrate that MLGA-CDRF-YOLO achieves an mAP@0.5 of 95.5%, a precision of 95.8%, and a recall of 88.5%, with only 3.41 M parameters and 14.8 GFLOPs, achieving a competitive and comprehensive balance between accuracy and computational cost. Furthermore, evaluation on the NEU-DET dataset confirms the model’s stable generalization performance across diverse detection tasks.

## 1. Introduction

To achieve the desired energy and lifetime of charged particle beams, the beams must travel along the designed trajectory, which is formed by a series of multipole magnets. To realize this designed trajectory, the multipole magnets need to be adjusted and installed to their designated positions, a process known as magnet collimation [1]. Current magnet collimation work primarily relies on tracking instruments and other detection devices, which only permit single-point measurement and suffer from low efficiency. To investigate fundamental questions such as the mechanism of electroweak symmetry breaking and the origin of mass, particle accelerators are being constructed at increasingly large scales. This trend extends the time span required for traditional magnet collimation methods. Employing computer vision techniques to recognize encoded target features on the surfaces of multipole magnets for collimation tasks offers advantages including rapid response and multi-point measurement capability [2]. However, during magnet collimation, the target pixel ratio of encoded targets [3] is merely 0.003% to 0.009%, making it difficult for conventional visual techniques to capture such fine-grained features. Moreover, during the identification of encoded targets, influences from different materials, shooting angles, and environmental factors [4,5] can lead to missed detections, thereby reducing recognition accuracy. The development of fast and accurate encoded target recognition technology is of significant importance for improving the efficiency of particle accelerator magnet collimation.

Research on object detection in computer vision is currently transitioning from traditional visual algorithms toward deep learning-based approaches. Specifically, object detection networks are mainly categorized into single-stage methods, two-stage methods, and Transformer-based methods [6]. Single-stage methods can directly predict bounding boxes and categories in a single forward pass and are renowned for their fast detection speed. Representative methods include the YOLO series, SSD, and RetinaNet [7]. Recent improvements to single-stage detectors have focused on enhancing small object detection through multi-layer feature optimization [8], global modeling modules for fine-grained feature discrimination, and Mamba-based feature pyramid enhancement [9]. Two-stage methods first generate candidate regions and then perform classification and regression, as exemplified by Faster R-CNN [10]. Subsequent work has improved detection consistency through class-agnostic non-maximum suppression combined with feature pyramid networks [11], and enhanced small object accuracy via optimized anchor boxes and region proposal parameters [12]. A comprehensive review chronicles this evolution from YOLOv1 to YOLOv12, highlighting incremental advances in speed, accuracy, and computational efficiency [13].

Recently, YOLO-based variants have addressed diverse detection challenges: multi-stage feature enhancement for general object detection [14], multi-scale defect detection in sewer pipelines [15], small object detection for UAVs under complex lighting and occlusion [16], and blurred vehicle detection in dynamic traffic environments [17]. YOLOv11 has demonstrated strong multi-class detection performance on high-resolution remote sensing imagery [18]. For small objects specifically, deep feature learning with multi-scale receptive fields and shallow-deep feature fusion has yielded significant gains [19].

Attention mechanisms have also been widely integrated into YOLO architectures to improve small object detection. Cross-spatial multi-head self-attention has been used to establish long-range contextual dependencies [20], and hierarchical Swin Transformer attention with shifted-window self-attention has enhanced global context modeling for small UAV detection [21]. Parameter-free attention combined with multi-scale feature pyramids has been employed to improve densely distributed small object detection while simultaneously reducing model parameters [22]. Recent YOLO iterations have further introduced NMS-free end-to-end training strategies [23] and improvements in bounding box prediction, cross-scale prediction, and feature extraction [24].

This paper proposes a lightweight multi-scale channel attention dynamic residual fusion module, which enhances deep attention modeling capability compared with other lightweight models. It reduces model parameters while maintaining detection performance and improves the ability to recognize extremely small encoded targets in the multipole magnet collimation context. Specifically:

(1) We propose the MLGA-CDRF-YOLO framework, a lightweight and high-performance small object detection model built upon YOLOv11s, which reduces computational cost while improving detection accuracy.
(2) We propose the C2f-CDRF module, which organically integrates a lightweight channel attention mechanism with a dynamic residual structure. This not only enhances the effectiveness of feature representation but also reduces the model’s parameter count and computational complexity, thereby strengthening the model’s learning capability and inference efficiency.
(3) We propose the MLGA module, which employs a dual-branch group attention design: A channel attention branch captures global channel-wise dependencies via pooling and depthwise separable convolution, while a multi-scale spatial attention branch uses parallel GhostConv layers at different scales; a ChannelShuffle operation facilitates information exchange across channel groups. This design effectively fuses multi-scale feature expressiveness with efficient channel modeling capability, thereby improving the model’s representational and generalization abilities for targets in complex scenes.
(4) By comparison with multiple YOLO variants, the MLGA-CDRF-YOLO achieves competitive mAP and precision on the encoded targets dataset with substantially reduced parameter count and computational cost, while maintaining stable generalization on the NEU-DET dataset.

## 2. Related Work

This paper presents lightweight improvements to the YOLOv11s model for the accelerator magnet collimation scenario, designing a target recognition algorithm tailored to this application.

### 2.1. YOLO-Based Object Detection Algorithm

YOLO series detectors decompose object detection into a single regression problem, directly predicting bounding boxes and class probabilities from full images in one forward pass. Their modular backbone–neck–head architecture allows independent optimization of feature extraction, multi-scale fusion, and task-specific prediction, making them well-suited for customization in domain-specific applications such as industrial inspection and scientific instrumentation.

### 2.2. Model Lightweighting Techniques

Lightweight network design pursues parameter and computation reduction without proportionally sacrificing accuracy. Two complementary strategies dominate: post-training compression, where knowledge distillation [25] transfers the representational capacity of a larger teacher model into a compact student; and structural simplification, which replaces costly operators with efficient alternatives such as depthwise separable convolution [26], Group Convolution [27], Ghost Convolution [28], and PConv [29]. A related modular redesign approach is RePack then Refine by Dong [30], which compresses high-dimensional vision features to accelerate Diffusion Transformer training, sharing a similar philosophy of inserting targeted modules into mature frameworks without full backbone redesign.

CBAM enhances feature representation through channel and spatial attention [31]. GhostNet reduces feature redundancy using inexpensive operations [28], while ShuffleNet promotes inter-group information exchange through channel shuffling [27]. However, these methods were not specifically designed for extremely small encoded targets in high-resolution images.

## 3. Method

This section details the proposed improvements to the backbone and attention modules of YOLOv11s, comprising the C2f-CDRF feature extraction module and the MLGA module. Both modules are designed as drop-in replacements that preserve compatibility with the YOLO architecture series.

### 3.1. Overall Architecture of MLGA-CDRF-YOLO

YOLOv11s serves as the baseline for this study. Among the five official scales, the “s” variant offers a favorable accuracy–speed trade-off while leaving room for structural optimization. A key limitation of the standard C3k2 block in YOLOv11s is its reliance on plain convolution for feature extraction: intermediate layers produce many near-duplicate feature maps, wasting computation and memory bandwidth. Moreover, the fixed local receptive field of plain convolution restricts long-range dependency modeling, which is particularly detrimental for detecting encoded targets that occupy as little as 0.003% of image pixels and whose contextual cues span large spatial distances [32].

**Figure 1:**
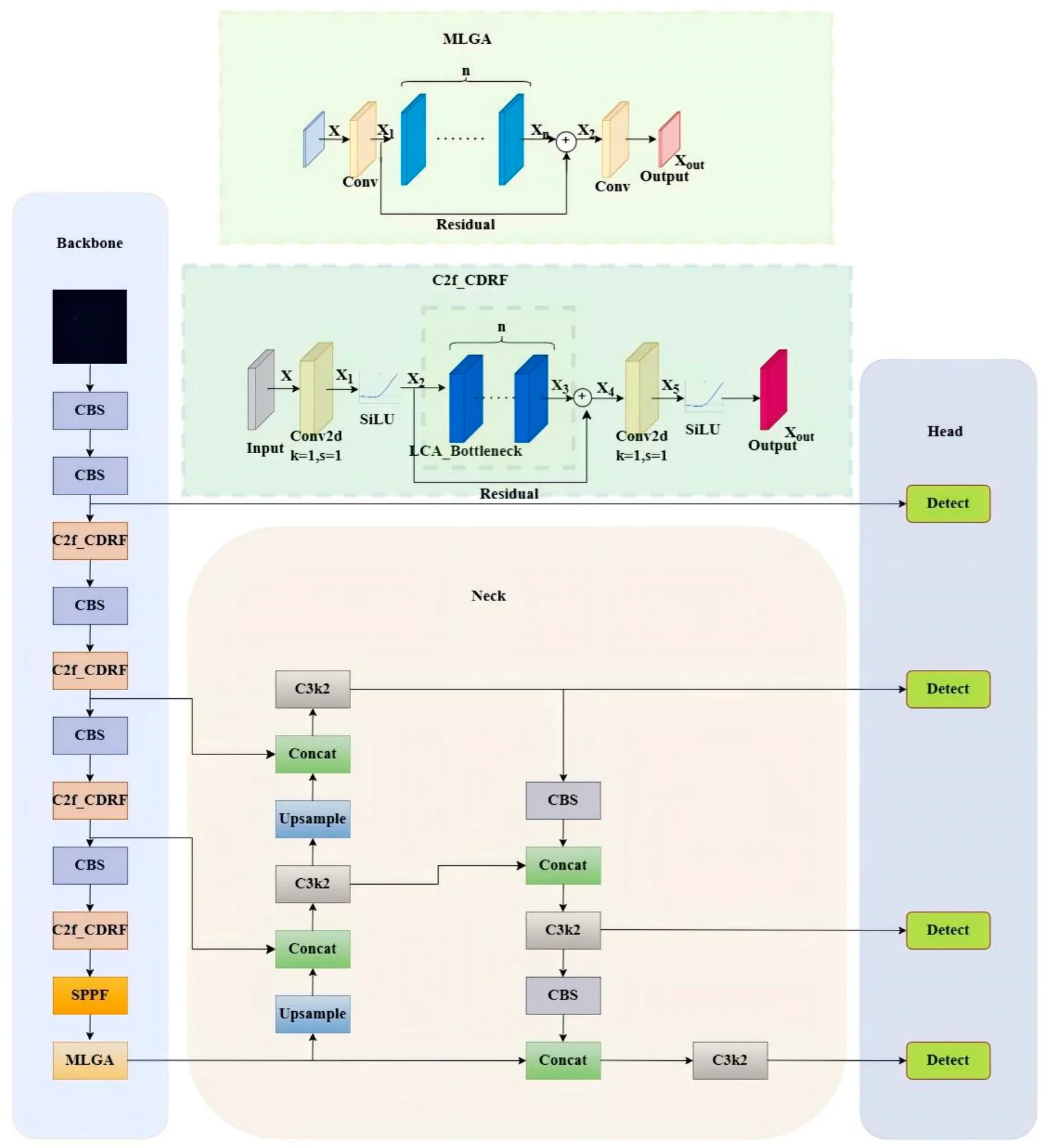
The network structure of the MLGA-CDRF-YOLO network.

This paper develops MLGA-CDRF-YOLO as a scenario-specific modular variant of YOLOv11s rather than a redesign of the overall YOLO framework. The simplified architecture of the MLGA-CDRF-YOLO network is shown in Figure 1. We redesign the bottleneck unit with a lightweight channel attention block, which substitutes depthwise separable convolution for standard convolution and integrates CBAM-based spatial attention, substantially cutting parameters and FLOPs. Stacking these blocks yields the LCA-Bottleneck, the building unit of the C2f-CDRF module. C2f-CDRF replaces the C3k2 block in both the backbone and neck of YOLOv11s, preserving multi-scale feature fusion while compressing model scale. Second, for the deepest network layers, we improve the C2PSA attention module by incorporating GhostConv-generated multi-scale spatial branches and a channel attention pathway, forming the MLGA module. This design strengthens global context aggregation with lower overhead than the original self-attention mechanism.

For the deep layers of the network, we improve Ghost Convolution and integrate it into the PSA block to form a structure named MSCA. Through GhostConv, a small number of intrinsic feature maps are generated, and multi-scale spatial convolution (MSCB) combined with a channel attention module is utilized for feature enhancement, achieving cross-channel and cross-spatial information interaction. On this basis, we construct the MSCA feature extraction module and embed it into the C2PSA layer of YOLOv11 to enhance global context modeling capability. Compared with the original C2PSA module that relies solely on standard convolution and self-attention mechanisms, the operational characteristics of GhostConv reduce parameter count and computational complexity, while multi-scale spatial perception and channel adaptive calibration effectively improve the model’s feature extraction capability and detection accuracy for multi-scale targets.

Targeting the characteristics of the collimation task, including the extremely small proportion of encoded targets, high-resolution input, and computational-efficiency requirements, we redesign and integrate them for extremely small target detection. MLGA combines parallel channel and multi-scale spatial branches with ChannelShuffle, while C2f-CDRF reduces computational costs through lightweight feature extraction and residual fusion.

### 3.2. LCA-Block

LCA-Block is a lightweight feature enhancement module that combines depthwise separable convolution, channel attention, and spatial attention mechanisms to improve feature expressiveness while reducing computational cost. In this module, we replace the ReLU [33] function with the SiLU [34] function. This is because different activation functions suit different application scenarios, and selecting the appropriate one is crucial for the performance and accuracy of deep learning models.

SiLU is a nonlinear activation function that has attracted considerable attention in the deep learning field in recent years. It is expressed as

(1) $$SiLU\left(x\right)=\frac{x}{1+e^{-x}}$$

Compared with ReLU, SiLU combines the non-saturating advantage of ReLU with the smooth gradient property of the Sigmoid function [35], adaptively regulating the output response according to the input signal. Its continuous gradient propagation mechanism suppresses the vanishing gradient problem and ensures stable backpropagation [28]. Based on these advantages, we select the SiLU function to activate feature maps extracted by depthwise separable convolution, which enhances feature distinctiveness and facilitates the capture of tiny targets and subtle variations in the data.

**Figure 2:**
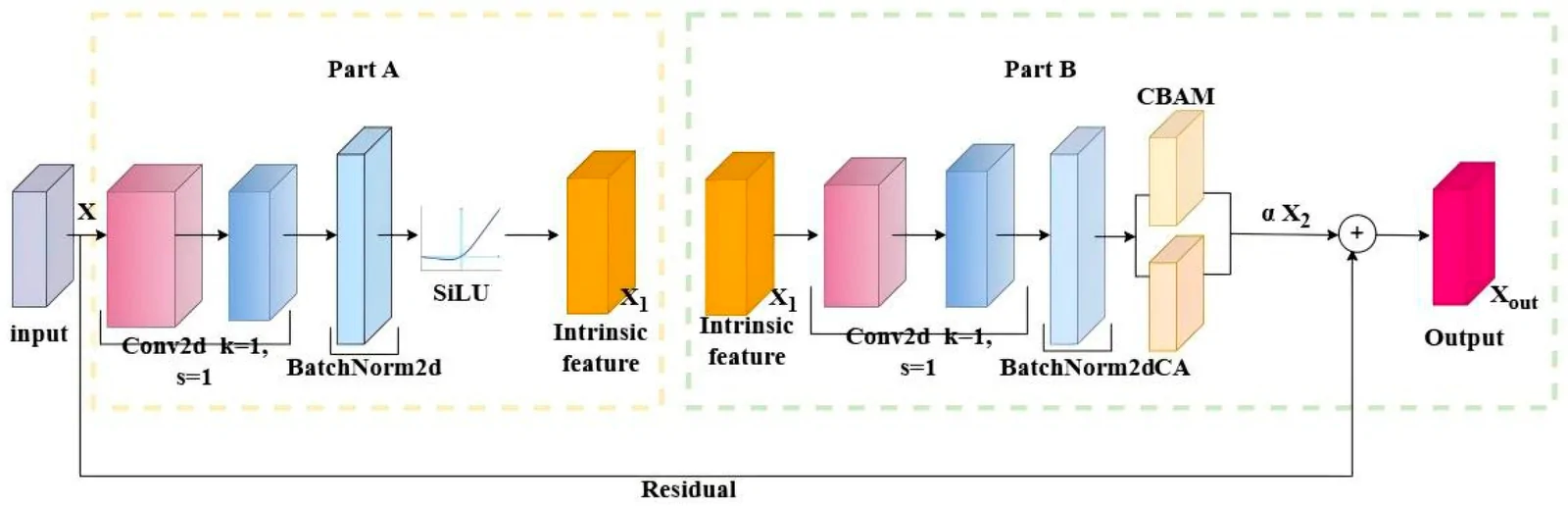
The network architecture of LCA-Block.

As shown in Figure 2, the input feature map is initially processed through the first part to generate fewer intrinsic feature maps, X_1,_ than standard convolution. Specifically, the input X first undergoes channel transformation via a 1 × 1 standard convolution, followed by batch normalization to stabilize the distribution, and then the SiLU activation function to introduce non-linear features, ultimately yielding the feature map denoted as X_1_. Subsequently, X_1_ passes through the second part, where linear operations are employed to generate redundant feature maps X_2_ that are highly similar to X_1_, which are then enhanced through Channel Attention and the CBAM module. Finally, a residual connection with the input X produces the output feature map X_out_.

(2) $$X_{1}=f_{1}\left(X\right)$$

(3) $$X_{2}=f_{2}\left(X_{1}\right)$$

(4) $$X_{out}=add(X,\;\alpha X_{2})$$

where *f*_1_ denotes the first-stage operation, which applies convolution, BatchNorm, and activation function processing to the input feature X to extract intrinsic feature maps. *f*_2_ denotes the second-stage operation, which further applies convolution, BatchNorm, and parallel processing of channel attention and CBAM to X_1_ to generate redundant feature maps. *α* denotes the scaling factor, experimentally determined to be 1.2 [32]. *add* denotes the residual connection between X and *α*X_2_.

### 3.3. LCA-Bottleneck

Standard convolution generates redundant feature maps during the feature extraction process, leading to an increased parameter count. Moreover, it is incapable of modeling spatial and channel features separately, making it difficult to capture finer-grained feature relationships [36]. To address this issue, this paper creates the LCA-Bottleneck module. This enables feature extraction while reducing computational cost, simultaneously improving feature representation.

**Figure 3:**
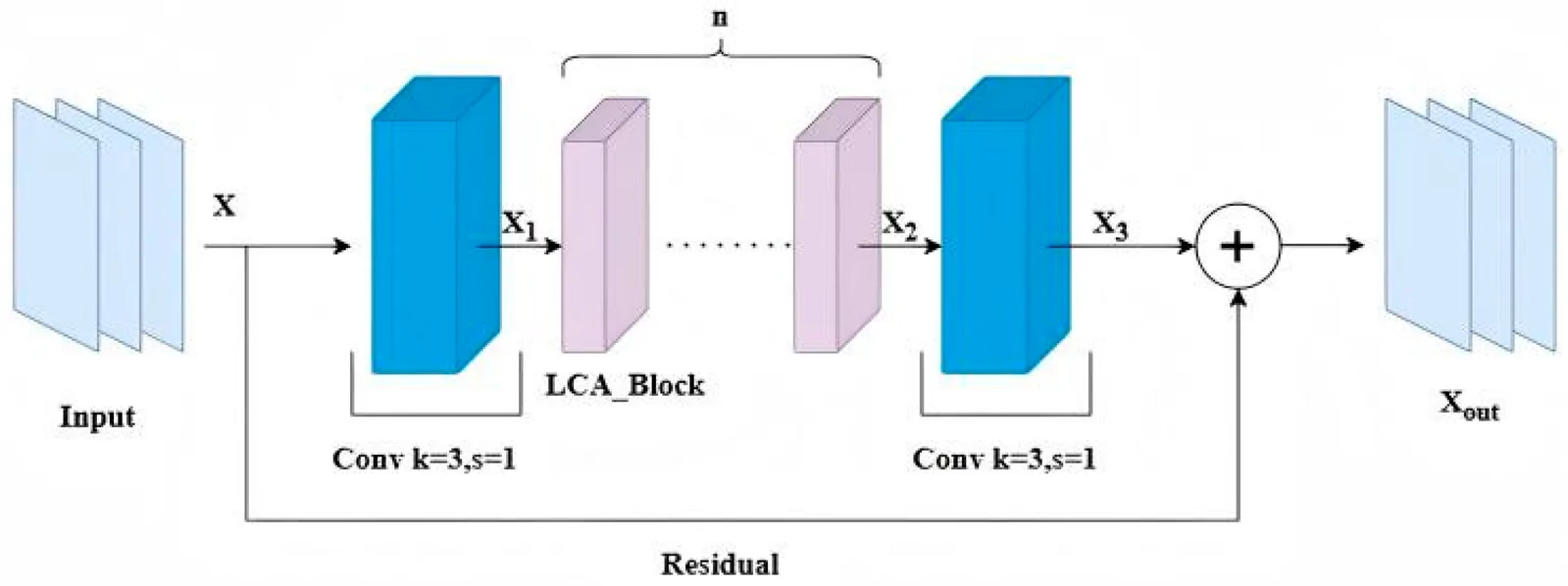
The network architecture of LCA-Bottleneck.

As shown in Figure 3, the input feature map X first passes through the first standard convolution layer to establish initial feature representation and extract low-level features. In this convolutional layer, the number of feature channels is expanded. The feature maps processed by the initial convolution are then fed into a sequence composed of *n* LCA-Block modules. This constitutes the core component of the LCA module, responsible for extracting key features through the local channel attention mechanism. Each LCA-Block adopts a lightweight design internally, enhancing features while reducing computational cost. Finally, the key feature maps processed by the *n* LCA-Block modules are connected residually with the input feature map X. This process integrates attention-enhanced feature information while preserving the integrity of the original information, alleviating the adverse effects of information loss caused by multiple convolutions, and yielding the output X_out_ of the LCA-Bottleneck structure.

(5) $$X_{1}=f_{1}\left(X\right)$$

(6) $$X_{2}=f_{2}\left(X_{1}\right)$$

(7) $$X_{3}=f_{3}\left(X_{2}\right)$$

(8) $$X_{out}=f_{4}\left(X,\;X_{3}\right)$$

where *f*_1_ denotes the first convolution operation on the input X. *f*_2_ denotes the *n* LCA-Block operations performed on the feature map X_1_. *f*_3_ denotes the second convolution operation. *f*_4_ denotes the residual connection between the input X and X_3_.

### 3.4. C2f-CDRF

**Figure 4:**
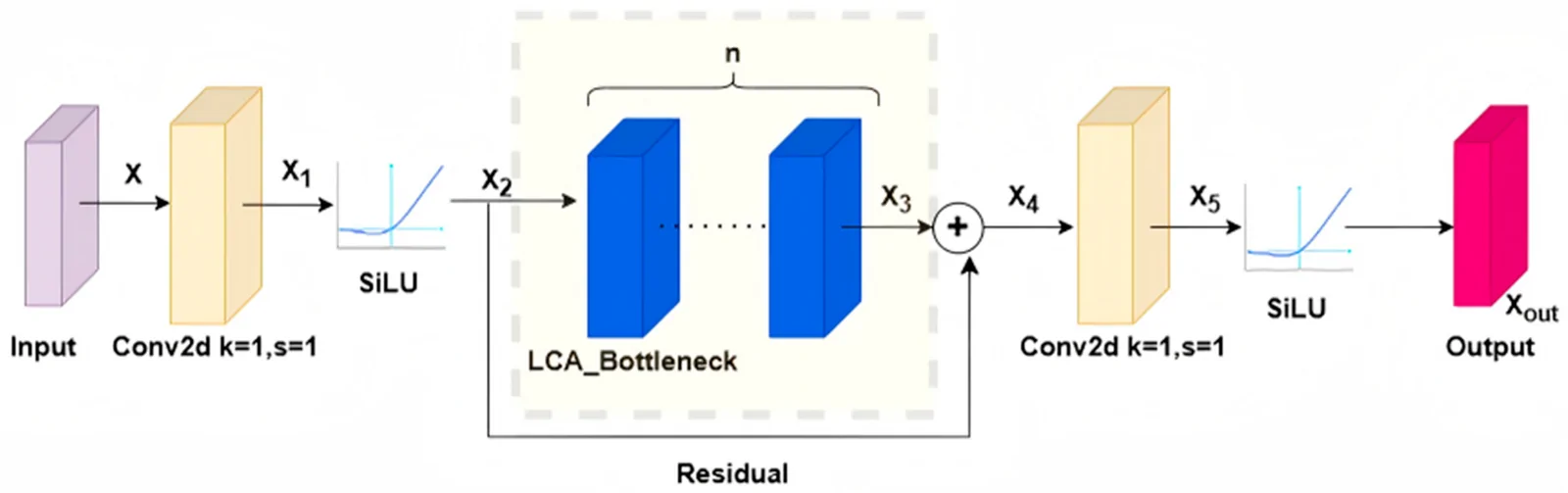
The network architecture of C2f-CDRF.

To optimize resource utilization during model computation and reduce the redundancy of feature maps generated by standard convolution that leads to resource waste, this paper redesigns the C2f feature extraction module using LCA-Bottleneck, reducing feature parameter count and computational complexity while enhancing feature representation, termed C2f-CDRF. As shown in Figure 4, we first reduce the number of channels through a 1 × 1 convolution to lower the computational load of subsequent operations, obtaining the feature map X_1_. The SiLU activation function is then applied to introduce non-linearity, yielding the feature map X_2_. We employ a feature extraction layer constructed from *n* LCA-Bottleneck modules to extract input features, obtaining the feature map X_3_. The feature map is then added to X_3_, derived through an identity mapping of the input feature map X along the channel axis, to preserve more spatial information and enhance model robustness. Finally, channel integration is performed via a 1 × 1 convolution operation, followed by the SiLU activation function to enhance feature expressiveness, generating the feature map X_out_.

(9) $$X_{1}=f_{1}\left(X\right)$$

(10) $$X_{2}=SiLU\left(X_{1}\right)$$

(11) $$X_{3}=f_{2}\left(f_{2}\left(\ldots f_{2}\left(f_{2}\left(X_{2}\right)\right)\right)\right)$$

(12) $$X_{4}=add(X_{2},\;X_{3})$$

(13) $$X_{5}=f_{1}\left(X_{4}\right)$$

(14) $$X_{out}=SiLU\left(X_{5}\right)$$

where *f*_1_ denotes a standard convolution operation, and *f*_2_ represents the feature extraction operation of LCA-Bottleneck. This paper employs the C2f-CDRF feature extraction module to obtain the new feature map X_out_. The C2f-CDRF module is applied to both the backbone and the neck, aiming to reduce computational complexity and parameter count while maintaining the feature extraction capability of the backbone network and the feature fusion capability of the neck.

In our design, the number of LCA-Bottleneck modules is set to *n* = 4 [31]. This choice stems from the structure of the base YOLOv11s model and maintaining consistent model depth constitutes the core principle of our experimental design. This enables us to conduct comparative analysis, ensuring that any observed performance improvements can be definitively attributed to the architectural superiority of the LCA-Bottleneck module, thereby effectively isolating the innovation of our proposed structure from factors such as model capacity expansion or increased computational complexity.

### 3.5. MLGA

The C2PSA module is located in the deep layers of the network, aiming to generate more discriminative high-level semantic feature maps, which play an important role in the model’s understanding capability and decision-making process. C2PSA fuses multi-scale feature information through cross-channel pyramid self-attention mechanisms, enhancing the model’s perception capability for complex scenes and fine-grained targets. This module can dynamically capture relationships between distant regions, breaking through the limitations of traditional fixed-window operations and strengthening the model’s global context modeling capability. Compared with structures that rely solely on local features, C2PSA exhibits stronger robustness and generalization ability when processing object detection tasks such as encoded points, significantly improving the model’s recognition effectiveness for tiny targets and overall scene understanding capability. Furthermore, due to its complex attention computation, the inference speed decreases compared with lightweight attention modules.

To address these issues, this paper introduces two branches: a lightweight channel attention branch and a multi-scale spatial attention branch, whose function is to extract global information. This paper also employs the LeakyReLU [37] activation function to enhance the model’s generalization capability and improve its stability.

The LeakyReLU function is an improved variant of the ReLU activation function, which can avoid the phenomenon of neuron “death” when computing negative input values. Its mathematical formula is expressed as

(15) $$LReLU\left(x\right)=\left\{\begin{array}{c} x\;if\;x>0\\ \alpha x\;if\;x\leq 0 \end{array}\right.$$

*α* is set to 0.2 [38]. In object detection tasks, the area of background regions is far larger than that of target regions. Therefore, the feature responses in the MLGA module are obtained by concatenating the features from the two branches and fusing them through a 1 × 1 convolution. These responses typically exhibit a number of activation states approaching zero or negative values. The SiLU activation function applies non-linear suppression to negative regions, causing negative responses that originally contain key discriminative contextual information to be ignored. The LeakyReLU function merely applies mild linear attenuation to negative responses, effectively maintaining the sign characteristics and relative magnitudes of the responses, thereby ensuring the integrity of fused features [39].

**Figure 5:**
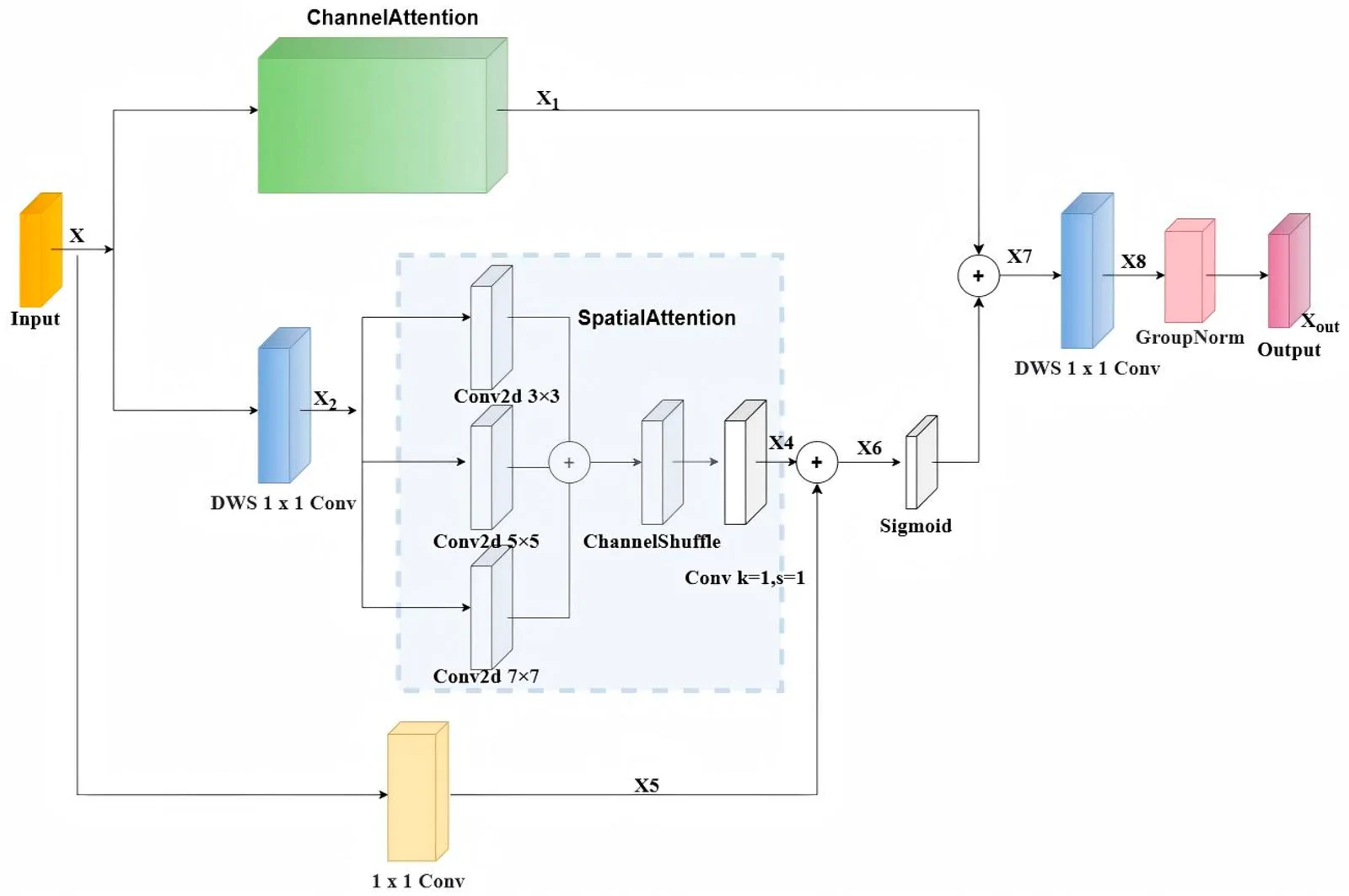
The network architecture of MSCB.

As shown in Figure 5, the MSCB processes the input feature map through parallel channel- and spatial-attention branches. The channel-attention branch aggregates global information using average and max pooling and then generates channel-enhanced features through depthwise separable convolutions and Sigmoid activation. In the spatial-attention branch, a 1 × 1 depthwise separable convolution first projects the input features, which are subsequently processed by parallel 3 × 3, 5 × 5, and 7 × 7 convolutions. The multi-scale responses are combined by element-wise addition, followed by channel shuffle and a 1 × 1 convolution. A projected shortcut is then added to the refined features before Sigmoid activation. Finally, the channel- and spatial-enhanced features are fused by element-wise addition and processed by a 1 × 1 depthwise separable convolution and group normalization to produce an output with the same dimensions as the input.

**Figure 6:**
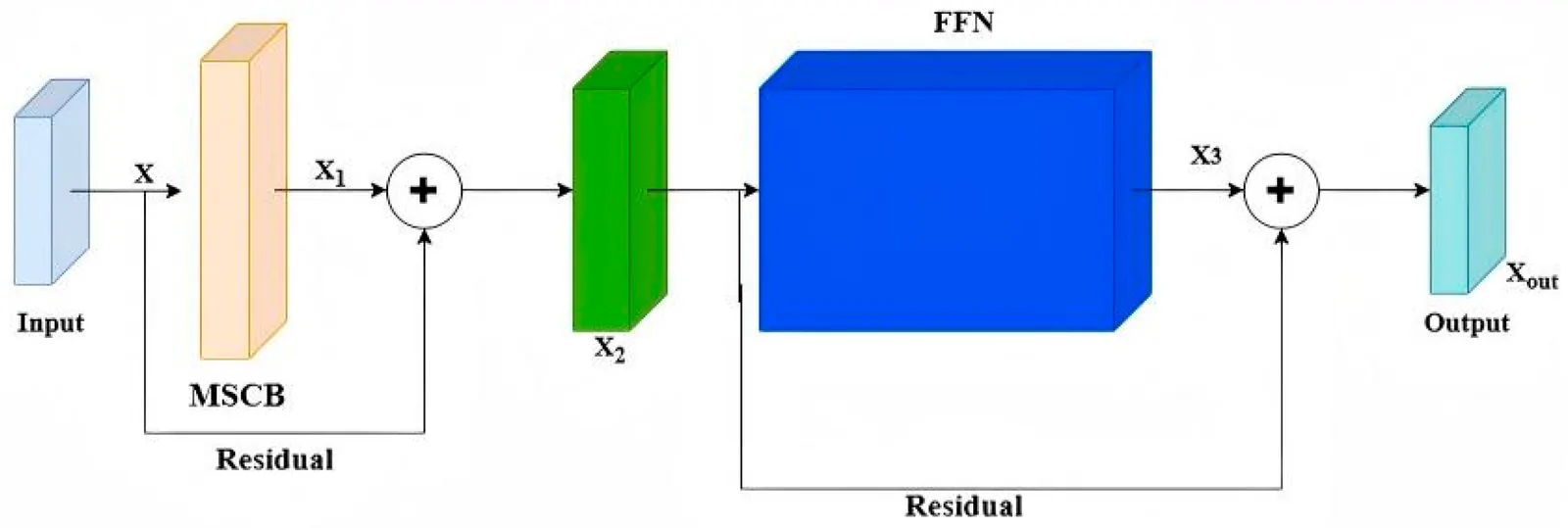
The network architecture of MSCA.

The MSCA module adopts a two-stage residual architecture, as shown in Figure 6. Given an input feature map X, the MSCB first captures complementary channel dependencies and multi-scale spatial context. Its output is added to X through the first residual connection to obtain X_2_. The fused feature is then passed through FFN. Finally, the FFN output is added to X_2_ through the second residual connection to generate X_out_. These residual connections preserve the original information and facilitate stable feature propagation.

**Figure 7:**
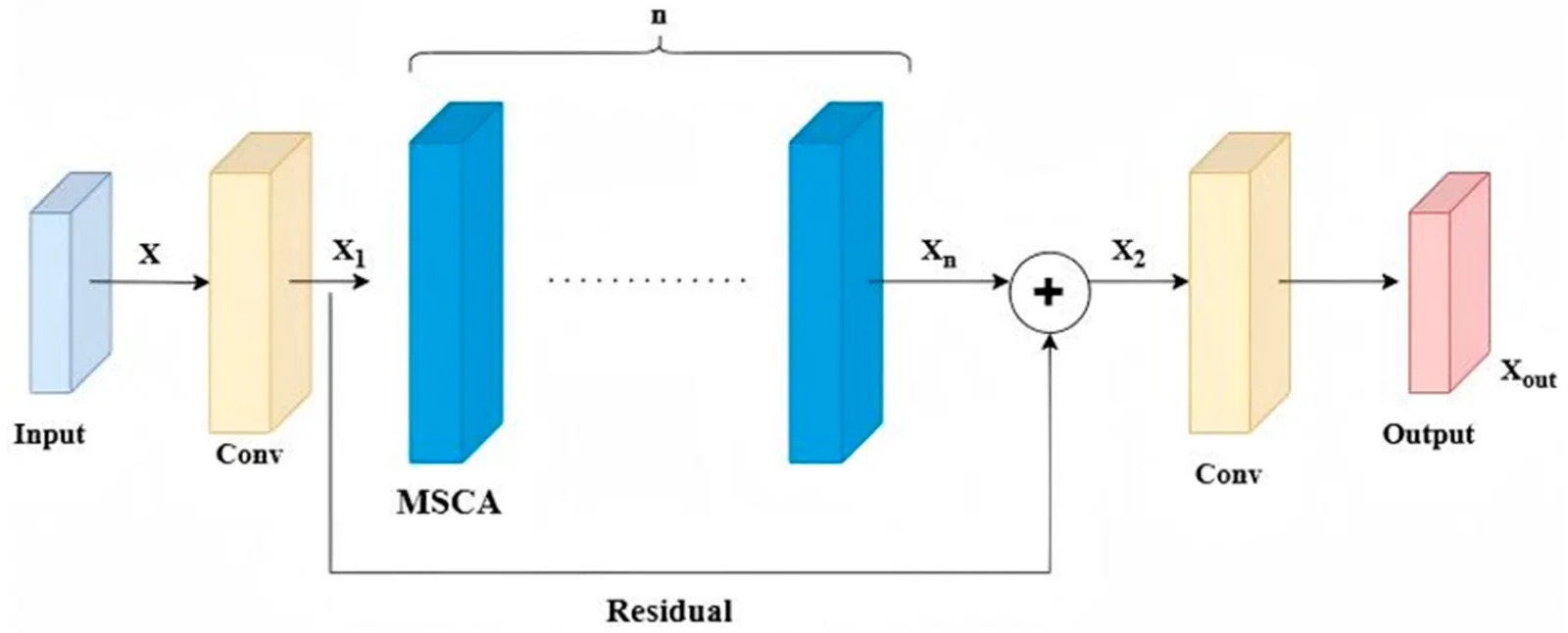
The network architecture of MLGA.

As shown in Figure 7, the input feature map X is first transformed by a standard convolution *f*_1_ to produce X_1_. X_1_ is then processed sequentially by *n* MSCA blocks, yielding the refined feature X_n_. In parallel, a long residual path transfers X_1_ directly to the fusion node. X_1_ and X_n_ are combined by element-wise addition to obtain X_2_, which is subsequently transformed by a second standard convolution *f*_3_ to generate X_out_.

(16) $$X_{1}=f_{1}(X)$$

(17) $$X_{n}=f_{2}\left(f_{2}\left(\ldots f_{2}\left(f_{2}\left(X_{1}\right)\right)\right)\right)$$

(18) $$X_{2}=add\left(X_{1},\;X_{n}\right)$$

(19) $$X_{out}=f_{3}\left(X_{2}\right)$$

where *f*_1_ denotes a standard convolution operation, *f*_2_ denotes the fused feature operation of the MSCA module, and *f*_3_ denotes a standard convolution operation.

## 4. Experiment

### 4.1. Experiment Environment

The experimental environment and training strategy of this model are summarized in Table 1. The label assignment strategy follows the anchor-free matching rule of YOLOv11: when the spatial distance between a prediction point and the center of a ground-truth box falls within a specific threshold range, the ground-truth box is assigned to that feature point. During training, feature points are dynamically filtered through centrality estimation and task collimation measurement. Each object is automatically assigned to one or more feature layers of different resolutions according to its scale, with small targets tending toward high-resolution feature maps and large targets tending toward low-resolution feature maps, achieving multi-scale collaborative prediction.

Comparative models are sourced from their official GitHub releases and evaluated under identical runtime conditions. For ablation studies involving structural modifications, all variant-specific settings such as learning rate, weight decay, optimizer, and data augmentation are held constant, ensuring that performance differences are attributable solely to architectural changes rather than training recipe variations. Every experiment—including ablation, comparative, and generalization tests—shares the same dataset split, hardware environment, batch size of 4, and training budget of 200 epochs.

### 4.2. Experimental Dataset Description

We validate our approach on two datasets with distinct characteristics. The primary benchmark is a dedicated encoded target dataset constructed for the multipole magnet collimation scenario, where targets occupy as little as 0.003% of image pixels. A secondary cross-domain evaluation is performed on NEU-DET, a public surface-defect dataset featuring six defect types under varying illumination and material conditions, to assess whether the proposed lightweight architecture generalizes beyond the collimation domain.

#### 4.2.1. Encoded Target Dataset

**Figure 8:**
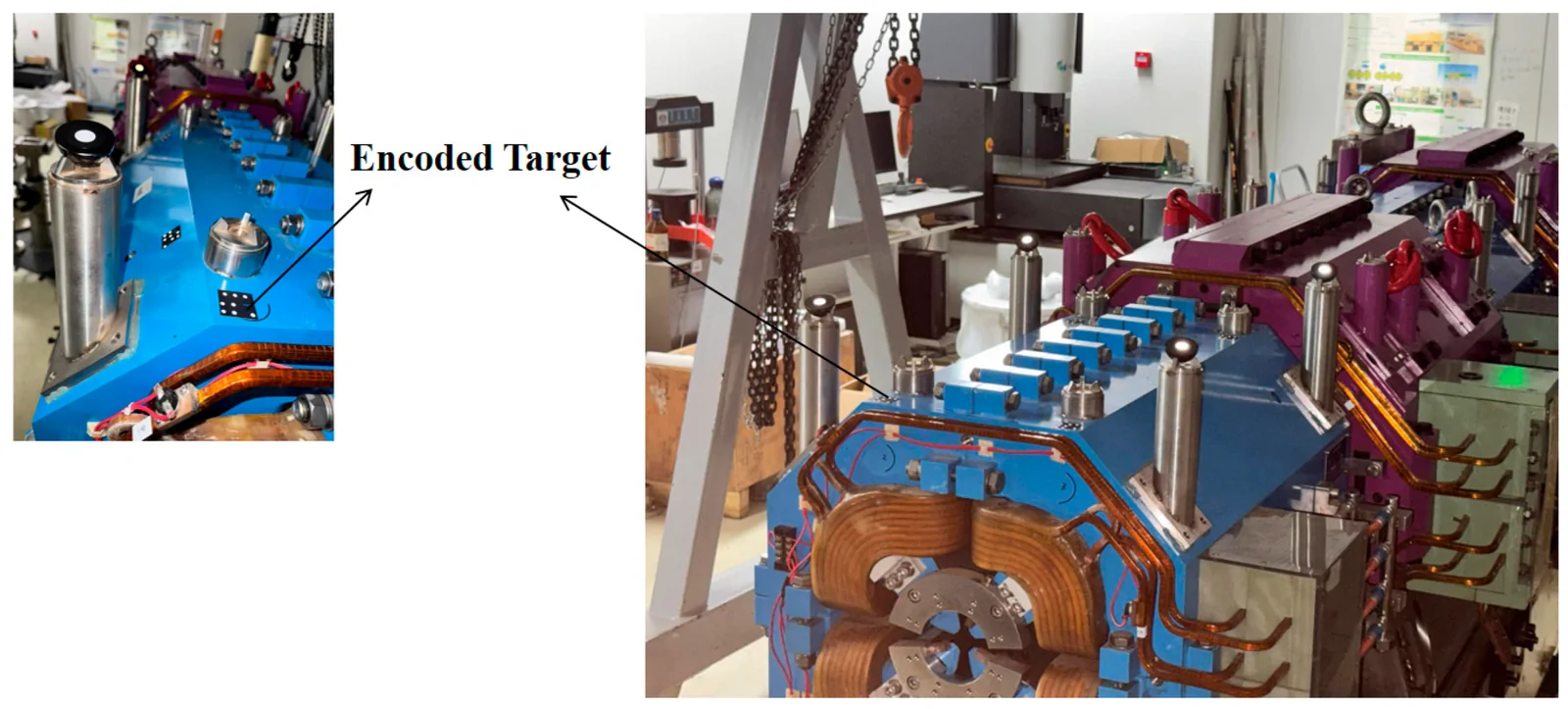
The experimental setting.

The experimental setting is shown in Figure 8. The dataset used in this study was collected by the team from the Institute of High Energy Physics, Chinese Academy of Sciences, at actual accelerator collimation sites, with the aim of improving the development and research of traditional encoded target recognition algorithms on multipole magnet surfaces. It comprises 350 high-resolution images with an average size of 5120 × 5120 pixels. Based on the original annotations, the encoded targets occupied approximately 0.003% to 0.009% of each 5120 × 5120 image; after resizing to 640 × 640 pixels, their corresponding areas were approximately 12 to 37 pixels. The images were collected under actual accelerator collimation conditions. Images were acquired using a single light source, with the camera positioned around the multipole magnet at a distance greater than 1 m from the encoded targets.

**Figure 9:**
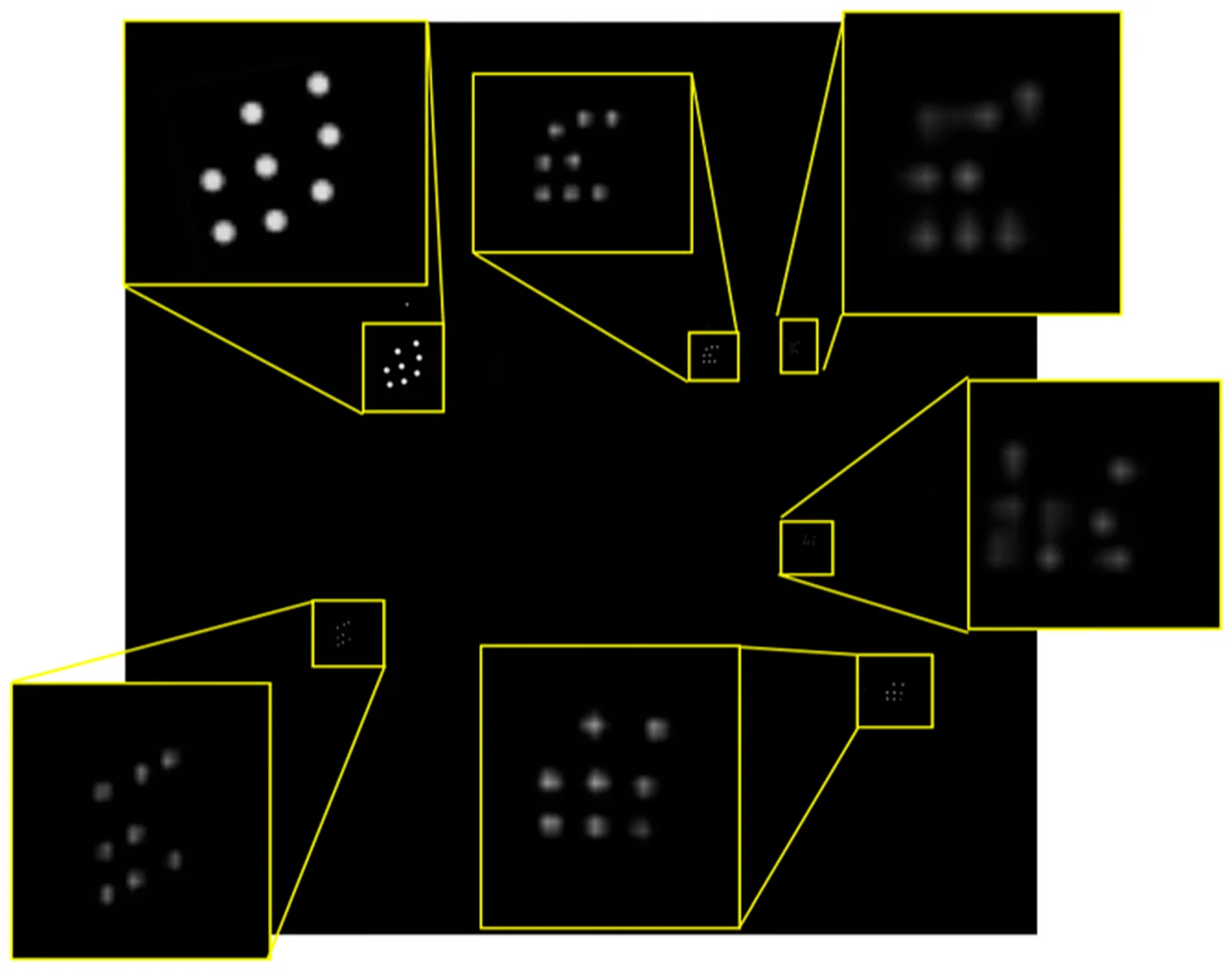
Encoded target dataset images.

The dataset was annotated using LabelImg and contains two categories, encoded frames and encoded points, with 6971 and 55,468 annotated instances, respectively. The majority of these are small-scale encoded targets, as illustrated in Figure 9. Because the number of images available for dataset construction was relatively limited, the dataset was split into training, validation, and test sets at an 8:1:1 ratio using stratified sampling based on category labels, to allocate as many images as possible to the training set and ensuring that the proportion of encoded frames and encoded points remained consistent across all subsets. This resulted in 280 training, 35 validation, and 35 test images.

In the process of creating the dataset, we perform feature enhancement on the training set images, including horizontal flip, Mosaic, HSV adjustment, translation and scale.

#### 4.2.2. NEU-DET

The NEU-DET [40] dataset was created by the team at Northeastern University, with the aim of studying the development and research of automatic detection algorithms for steel plate surfaces. The dataset contains six typical defect categories on steel plate surfaces: crazing, scratch, patch, pitted surface, rolled-in scale, and inclusion. It comprises 1800 steel plate surface defect images and serves as a standard dataset in the field of steel surface defect detection, suitable for rapid validation and comparative experiments of object detection algorithms. The dataset was split into training, validation, and test sets at an 8:1:1 ratio using stratified sampling based on the six defect categories, yielding 1440 training, 180 validation, and 180 test images with consistent class proportions across all subsets.

### 4.3. Experimental Metrics

The evaluation metrics employed in this paper are consistent with those adopted in most studies [41,42], including Precision, Recall, AP, and mAP, with mAP serving as the primary evaluation criterion. The definitions of these metrics are as follows:

(20) $$Precision=\frac{TP}{\left(TP+FP\right)}$$

(21) $$Recall=\frac{TP}{\left(TP+FN\right)}$$

(22) $$AP=\int_{0}^{1}p\left(r\right)\;dr$$

(23) $$mAP=\frac{1}{n}\sum_{I=1}^{n}{AP}_{i}$$

where *TP* denotes the number of true positives, *FP* denotes the number of false positives, and *FN* denotes the number of false negatives. *p*(*r*) denotes the precision–recall curve, and *n* denotes the number of categories. *AP* quantifies the detection accuracy for a single category, while *mAP* averages the detection accuracy across all categories. In this study, mAP@0.5 denotes the mean AP calculated at an IoU threshold of 0.50, whereas mAP@0.5:0.95 denotes the mean AP averaged over IoU thresholds ranging from 0.50 to 0.95 with a step size of 0.05. Both metrics are reported to evaluate detection accuracy under different localization criteria.

Parameter count is used to evaluate the number of parameters in the model, representing the total number of parameters during the training process. GFLOPs is an important metric for measuring model computational complexity, representing the total number of floating-point operations executed during model computation. Lower GFLOPs values indicate reduced computational complexity and resource requirements.

FPS reflects the model’s capability to process images per unit time, serving as an important metric for evaluating inference speed and real-time performance. It should be emphasized that inference latency is primarily influenced by model architecture.

All FPS measurements were conducted on an NVIDIA GeForce RTX 5070 Ti GPU using PyTorch 2.7.0 and CUDA 12.8. The input images were resized to 640 × 640 pixels, and the inference batch size was set to 1. Before timing, 5 warm-up iterations were performed to eliminate initialization overhead. Subsequently, 350 images were processed, and CUDA synchronization was performed immediately before and after timing. FPS was calculated as the number of processed images divided by the total elapsed time.

The reported FPS excludes image preprocessing, data loading, non-maximum suppression, and result visualization. The experiment on the encoded target dataset was independently repeated five times, and the mean and standard deviation were reported. All compared models were evaluated using the same hardware, software environment, input resolution, batch size, precision mode, and timing procedure.

### 4.4. Training Behavior Analysis

**Figure 10:**
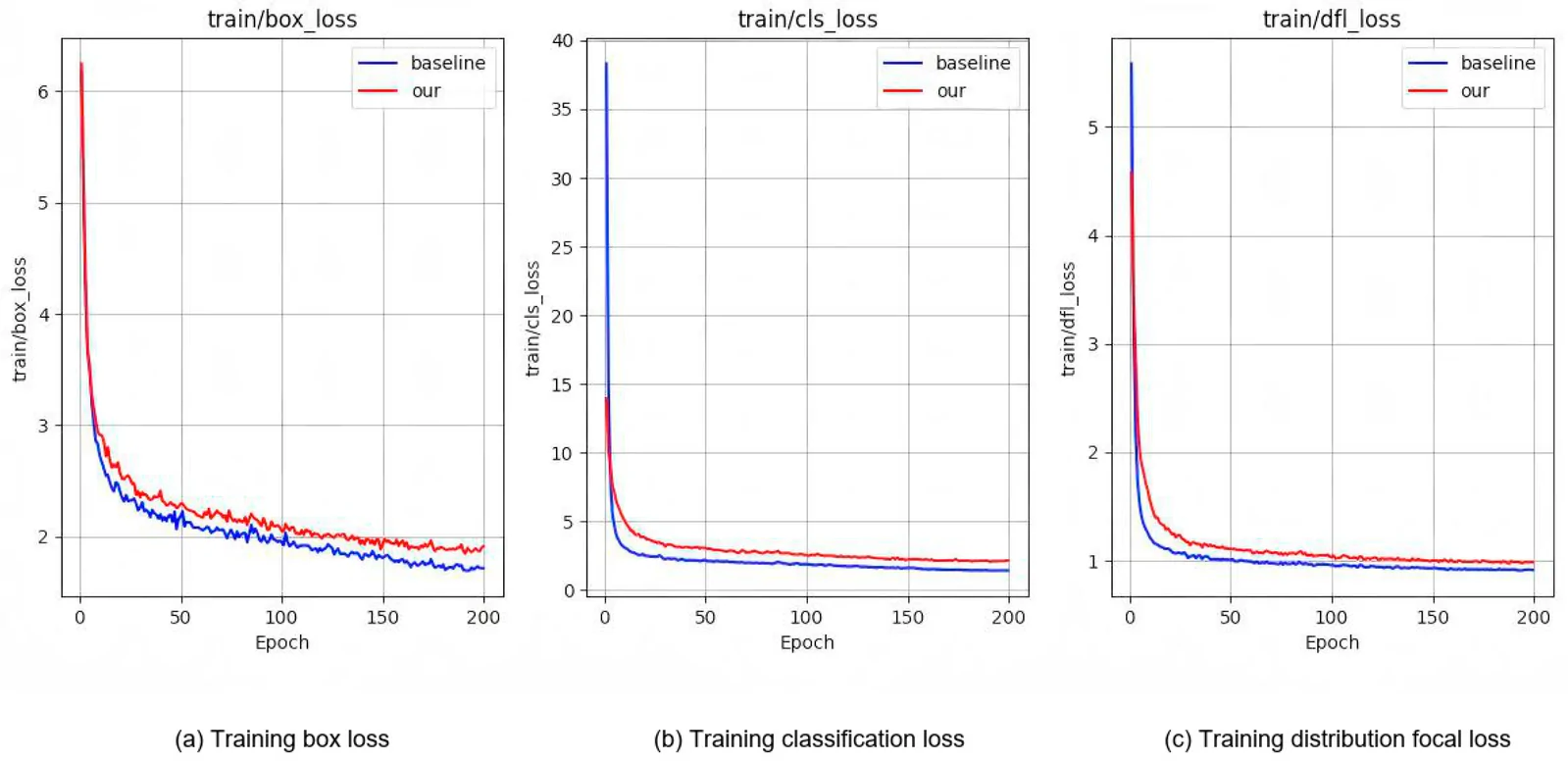
Training loss convergence curves.

By analyzing the training and validation loss curves, this paper interprets the model training dynamics. As shown in Figure 10, the training loss of MLGA-CDRF-YOLO decreases more steeply than that of YOLOv11s and reaches a lower value at the end of training. Under the reported training setting, these observable changes indicate faster convergence and a lower final training loss.

**Figure 11:**
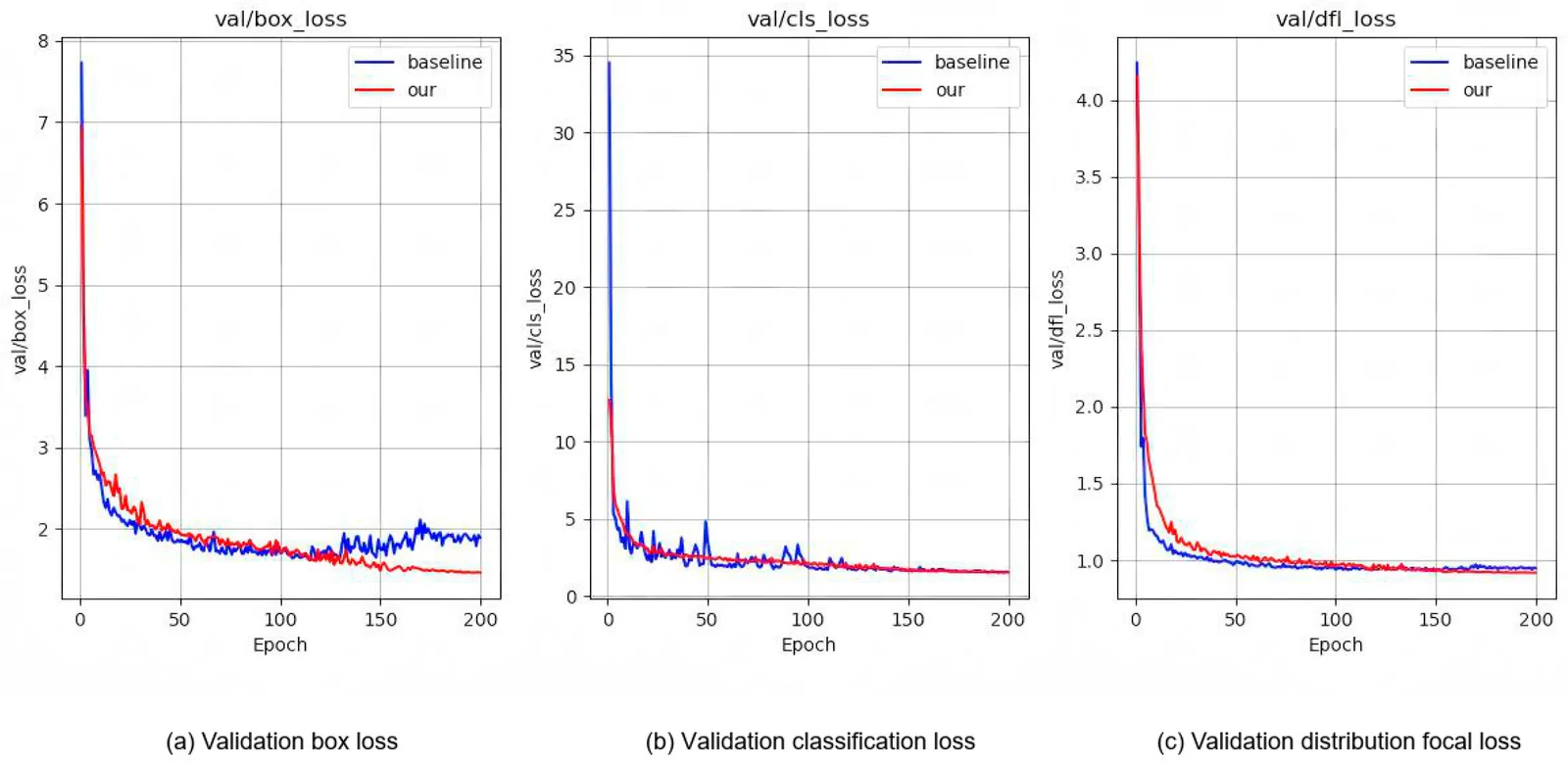
Validation loss convergence curves.

The validation loss curve comparison presented in Figure 11 shows that both MLGA-CDRF-YOLO and YOLOv11s exhibit high validation loss values during the early training stage. MLGA-CDRF-YOLO shows a larger initial loss peak, after which its validation loss decreases and remains lower than that of YOLOv11s for most of the remaining epochs.

### 4.5. Ablation Experiments

#### 4.5.1. Conduct Ablation Experiments on *α* and *n*

We conducted an ablation study to evaluate the effects of the residual-connection coefficient α and the number of module repetitions n on detection performance.

As shown in Table 2, *α* = 1.2 consistently outperformed *α* = 0.5 and 1.0 and achieved slightly better results than *α* = 1.4. Within the *α* = 1.2 group, increasing *n* from 1 to 4 improved all detection metrics, while increasing the parameter count by 0.09 M and causing a moderate decrease in FPS. Therefore, *α* = 1.2 and *n* = 4 were selected as the best-performing configurations in these experiments.

#### 4.5.2. Conduct Ablation Experiments on the Encoded Target Dataset

To evaluate the importance of each module in MLGA-CDRF-YOLO, this paper progressively introduces each module into the baseline model YOLOv11 to verify its effectiveness. The encoded target dataset is used for ablation experiments. Table 3 presents the impact of introducing each module on the evaluation metrics.

Although the Baseline + C2f-CDRF model achieves the lowest parameter count and computational cost among all models, its detection accuracy does not reach the optimal level. In contrast, our model achieves mAP@0.5 of 95.5% and a Precision of 95.8%, surpassing the other three models. On the other hand, regarding the Recall metric, our model is comparable to the Baseline + MLGA model and higher than the other two models. Finally, in terms of inference speed, the inference speed of our model is lower than that of the baseline + C2f-CDRF and baseline + MLGA model, but the gap with the other two models is not substantial, still maintaining efficient inference. Moreover, compared with the baseline model, our model reduces computational cost in terms of parameter count and computational cost, while improving mAP@0.5 by 2.3%.

**Figure 12:**
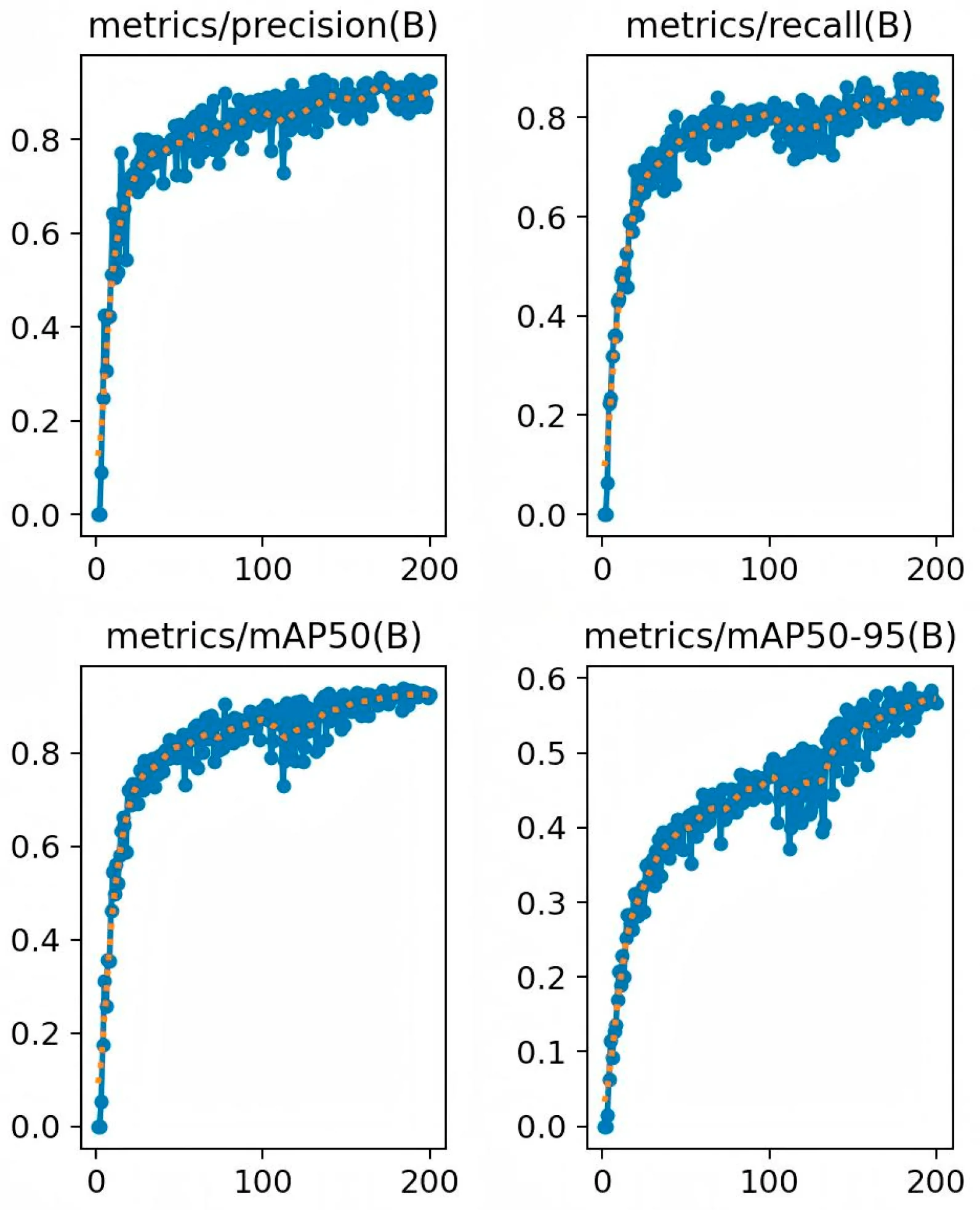
The training curves of MLGA.

Notably, the Baseline + MLGA configuration exhibits a Precision decrease from 94.4% to 86.9% and a slight mAP@0.5 decline from 93.2% to 92.9%, despite achieving the highest Recall among the single-module variants. As shown in Figure 12, analysis of the MLGA training curves reveals that the validation Precision oscillates substantially during the final epochs, indicating training instability introduced by the group attention mechanism with ChannelShuffle when applied in isolation. Specifically, the multi-scale spatial attention branch with parallel GhostConv layers expands the receptive field to capture more candidate regions, which improves Recall but simultaneously generates more false positives that reduce Precision. Without the C2f-CDRF module to first enhance and compress feature representations, the abrupt feature group splitting and ChannelShuffle operation disrupt feature coherence. When both modules are combined in the full model, C2f-CDRF pre-processes features through channel attention and dynamic residual fusion, providing more discriminative representations that allow MLGA to operate effectively, thereby restoring Precision to 95.8% while maintaining high Recall.

**Figure 13:**
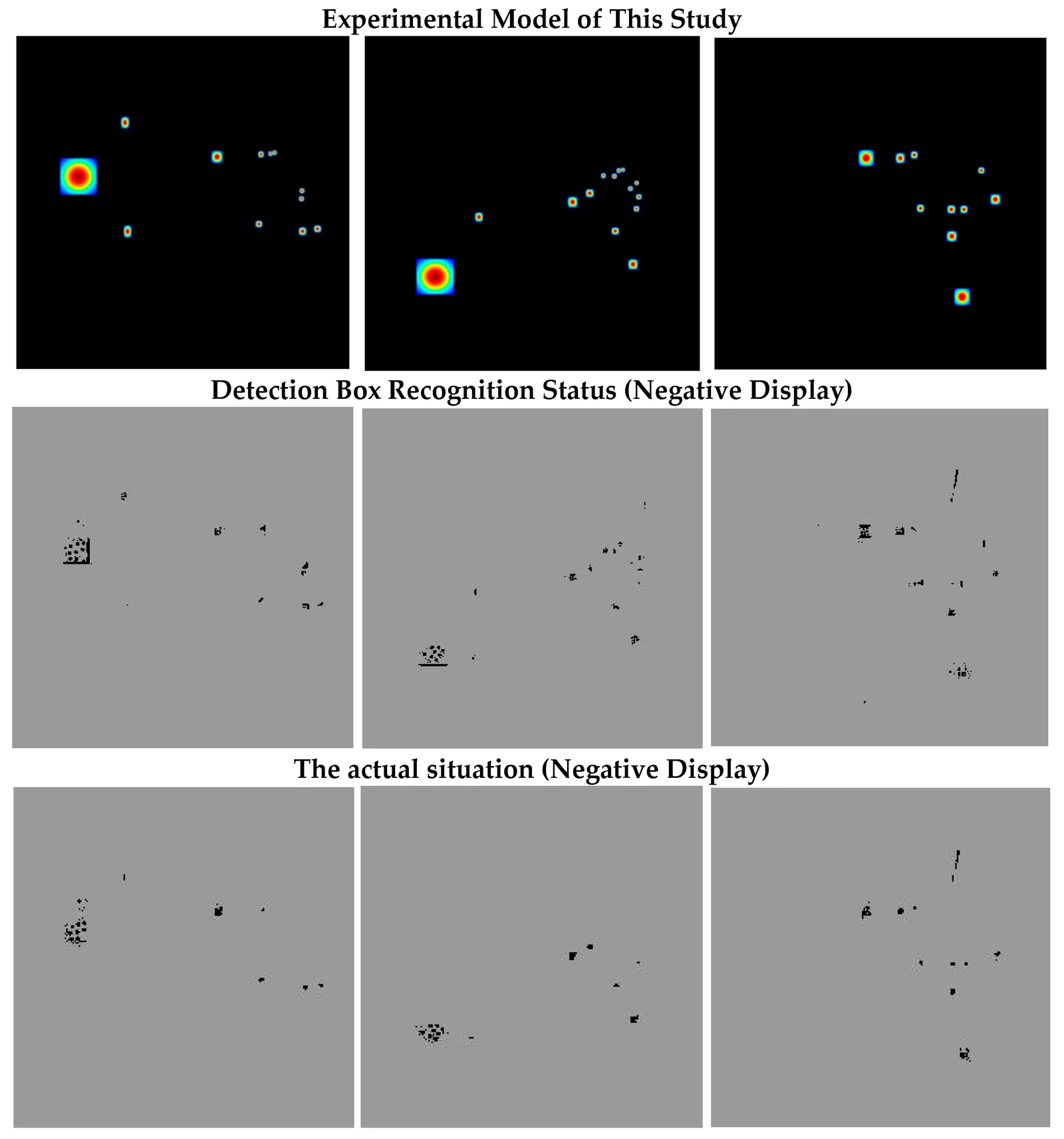
Local heatmap of the encoded target dataset.

Additionally, to more intuitively demonstrate the enhancement effects of the improved model, this paper employs gradient-weighted class activation mapping to generate localized heatmaps. This method computes the gradient of the target class score with respect to the last convolutional feature map, extracts channel weights through global average pooling, and subsequently fuses the feature maps in a weighted manner to localize key discriminative regions. As shown in Figure 13, for clearer visualization, the images were displayed in negative mode. The improved network is capable of accurately localizing extremely small encoded point regions in high-resolution images, avoiding the dispersion of attention to background walls, pipelines, and other interfering objects.

### 4.6. Comparison with Other Methods

To verify the improvement effects of the model, its detection performance was compared with other models. Table 4 lists the comparison results. All models were trained using the same dataset split, input resolution, batch size, number of epochs, optimizer, learning rate, and data-augmentation settings.

In the magnet collimation scenario, missed detections prevent the magnet at that position from completing collimation localization, necessitating camera angle readjustment and re-imaging, which severely degrades collimation efficiency. Therefore, recall serves as the critical metric for assessing collimation practicality. Compared with mainstream YOLO series models, our method achieves a recall of 88.5%. Although RT-DETR and GS_YOLO achieve higher recall on this dataset, our model attains the highest mAP@0.5 and precision among all compared methods with only 3.41 M parameters and 14.8 GFLOPs. Similarly, our recall reaches 88.5%, contributing to reliable detection capability in real collimation scenarios at reduced computational cost. Other models in the YOLO series continuously iterate and optimize based on the original architecture in pursuit of superior detection performance, yet their lightweight design and accuracy balance in terms of parameter count and computational complexity control remain suboptimal. Although the RT-DETR model achieves the highest recall among these models, its parameter count and computational cost far exceed those of our model. In contrast, our model achieves 95.5% mAP@0.5 with 3.41 M parameters and 14.8 GFLOPs computational cost, striking a balance between mAP@0.5 and efficiency.

### 4.7. Generalization Comparison

To evaluate the generalization capability of our method, comparative experiments are conducted on the NEU-DET dataset. All models were trained using the same dataset split, input resolution, batch size, number of epochs, optimizer, learning rate, and data-augmentation settings.

As shown in Table 5, our method achieves competitive detection performance while reducing model parameter count and computational overhead. Compared with YOLOv5s, model parameters are reduced by 51.4% and computational cost by 6.3%; compared with YOLOv6s, parameters are reduced by 81.6% and computational cost by 67.3%; compared with YOLOv7s, parameters are reduced by 90.8% and computational cost by 86%; compared with YOLOv8s, parameters are reduced by 69.3% and computational cost by 48.6%; compared with YOLOv9s, parameters are reduced by 52.4% and computational cost by 44.6%; compared with YOLOv10s, parameters are reduced by 57.5% and computational cost by 39.6%; compared with YOLOv11s, parameters are reduced by 63.8% and computational cost by 30.5%. It is worth noting that, on the NEU-DET dataset, the mAP@0.5 of the proposed method is only 0.2% lower than that of YOLOv9s, and its recall is only 1.0% lower than that of YOLOv8s. This pronounced asymmetry between the reduction in computational cost and the slight performance degradation indicates that our lightweight design mitigates the adverse impact of accuracy decline. Compared with YOLOv5s, our method achieves higher mAP@0.5 and recall while operating at lower computational cost, exhibiting a favorable balance between mAP@0.5 and computational burden. Through network architecture optimization, we control model scale while maintaining high feature extraction capability. Experimental results demonstrate that for steel surface defect detection tasks, our method achieves a balance among mAP@0.5, recall, and computational cost.

### 4.8. Experiments Repeated on the Encoded Target Dataset

To verify that the model’s performance was stable rather than the result of a single favorable run, we independently repeated the experiments on MLGA-CDRF-YOLO using different random seeds, as shown in Table 6.

The model achieved average mAP@0.5, mAP@0.5:0.95, precision, and recall values of 95.46%, 68.00%, 95.74%, and 88.50%, respectively. The corresponding standard deviations ranged from 0.21 to 0.32, indicating limited performance variation under the same parameter settings. In particular, Seed = 2 produced the highest values for all four accuracy metrics, whereas Seed = 1 produced the lowest; nevertheless, the differences between the runs remained small. Moreover, the average inference speed was 92.46 ± 0.76 FPS, demonstrating consistent computational efficiency across repeated runs. Overall, these results indicate that MLGA-CDRF-YOLO exhibits stable performance.

Nevertheless, the relatively small number of independent test images may limit the statistical power of the evaluation and the generalizability of the conclusions. Future work will focus on collecting a larger and more diverse dataset under additional operating conditions.

## 5. Discussion

This study proposes the MLGA-CDRF-YOLO framework for encoded target recognition tasks in accelerator magnet collimation, which improves upon the YOLOv11s baseline model for encoded target recognition scenarios. Experiments on the encoded target dataset demonstrate that MLGA-CDRF-YOLO achieves lightweight improvements compared with the YOLOv11s baseline model, with mAP@0.5 improved by 2.3%, model parameter count reduced by 64%, and computational complexity decreased by 30%. It maintains detection accuracy while achieving the objective of model lightweighting. Meanwhile, generalization experiments conducted on the NEU-DET dataset, through comparison with other models, validate the generalization capability of our model.

In future work, we plan to construct more comprehensive and diverse encoded target datasets that encompass different measurement scenarios in multipole magnet collimation. We will also pursue further lightweight optimization to maintain efficient inference under varying computational conditions. Additionally, we will validate the generalization of the proposed model in non-multipole magnet collimation scenarios.

## 6. Conclusions

This study develops the C2f-CDRF feature extraction module, aiming to reduce model parameter count and computational complexity while enhancing feature extraction capability. Concurrently, an improved C2PSA module named MLGA is proposed, which can dynamically capture relationships between distant regions to strengthen the model’s global context modeling capability. Compared with other object detection models, our model achieves a balance between lightweight design, mAP@0.5, and generalization performance.

This paper validates the effectiveness of the improved method, which combines lightweight multi-scale group attention with channel dynamic residual fusion, realizing a favorable balance between mAP@0.5 and computational cost.

## Figures and Tables

**Table 1:** Experimental environment and training hyperparameters.

**Hardware**	CPU	AMD Ryzen 9 8940HX (AMD, Santa Clara, CA, USA)
	GPU	NVIDIA GeForce RTX 5070 Ti (NVIDIA, Santa Clara, CA, USA)
**Software**	OS	Windows 11
	PyTorch	2.7.0
	CUDA	12.8
	Python	3.10.19
**Data Augmentation**	Mosaic	1.0
	HSV_H (±)	0.015
	HSV_S (±)	0.7
	HSV_V (±)	0.4
	Horizontal Flip	0.5
	Translation	0.1
	Scale	0.5
**Training Setup**	Input Size	640 × 640
	Baseline Pretrained Weight	Yolov11s.pt
	Early Stopping Patience	50
	EMA	Enabled
	SyncBN	No
	LR Schedule	cosine annealing
	Optimizer	Adam
	Initial Learning Rate	0.0001
	Final Learning Rate Ratio	0.01
	Weight Decay	0.0005
	Momentum	0.937
	*β*_2_	0.999
	Batch Size	4
	Epochs	200
	Warmup Epochs	5.0

**Table 2:** Ablation experiments on *α* and *n*.

*α*	*n*	mAP@0.5	mAP@0.5:0.95	Recall	Param (M)	GFLOPs	FPS
0.5	1	80.7	39.7	71.0	3.32	14.2	95.7
	2	82.1	42.2	68.4	3.35	14.4	94.2
	3	82.2	39.5	73.0	3.38	14.6	93.4
	4	80.2	40.2	67.2	3.41	14.8	93
1.0	1	82.0	42.0	69.0	3.32	14.2	95.6
	2	81.8	41.1	69.4	3.35	14.4	94.4
	3	81.6	40.4	71.2	3.38	14.6	93.7
	4	81.2	39.8	70.1	3.41	14.8	92.2
1.2	1	92.8	63.8	84.6	3.32	14.2	95.8
	2	93.7	65.2	85.9	3.35	14.4	94.3
	3	94.6	66.7	87.0	3.38	14.6	93.4
	4	95.5	68.0	88.5	3.41	14.8	93
1.4	1	92.1	63.2	84.2	3.32	14.2	95.4
	2	93.2	64.8	84.9	3.35	14.4	94.4
	3	94.0	66.0	86.8	3.38	14.6	93.5
	4	95.1	67.5	88.0	3.41	14.8	93.1

**Table 3:** Ablation experiments conducted on the encoded target dataset.

Method	mAP@0.5	mAP@0.5:0.95	P	R	Param (M)	GFLOPs	FPS
Baseline	93.2	54.3	94.4	83	9.41	21.3	66.67
Baseline + C2f-CDRF	94.1	64.3	94.1	86.7	2.73	14.3	94.5
Baseline + MLGA	92.9	58.3	86.9	87.2	3.34	14.8	93.6
Our	95.5	68	95.8	88.5	3.41	14.8	93

**Table 4:** Comparison with Other Methods on the Encoded Target Dataset.

Method	mAP@0.5	mAP@0.5:0.95	Recall	Param (M)	GFLOPs	FPS
RT-DETR	94.9	56.1	90.7	19.8	57	42.7
GS_YOLO	93.47	57.63	91.3	5.16	10.7	67.43
YOLOv5s	84.73	51.25	69.7	7.02	15.8	82.3
YOLOv6s	87.6	52.3	71.4	18.5	45.3	60.5
YOLOv7s	65.33	30.02	64.3	37.22	105.3	78.3
YOLOv8s	87	50.9	79.2	11.12	28.8	41
YOLOv9s	65.5	29	65.8	7.16	26.7	64
YOLOv10s	83.5	51.6	73.9	8.03	24.5	65.34
YOLOv11s	93.2	54.3	83	9.41	21.3	66.67
Our	95.5	68	88.5	3.41	14.8	93

**Table 5:** Generalization Comparison on the NEU-DET Dataset.

Method	mAP@0.5	mAP@0.5:0.95	Recall	Param (M)	GFLOPs	FPS
RT-DETR	65.2	35.9	61.3	19.8	57	125.4
GS_YOLO [32]	74	40.1	71.1	5.16	10.7	81.96
YOLOv5s [32]	58.3	37.4	49.4	7.02	15.8	125
YOLOv6s [43]	73.6	41.3	68.7	18.5	45.3	50.14
YOLOv7s [44]	72.2	39.54	69.2	37.22	105.3	66.43
YOLOv8s [32]	74	39.7	74.6	11.12	28.8	77.51
YOLOv9s [45]	77.5	41.8	68.1	7.16	26.7	34.6
YOLOv10s [23]	71.9	38.9	66	8.03	24.5	56.49
YOLOv11s [46]	75.7	40	69.8	9.41	21.3	63.29
Our	77.3	42.1	73.6	3.41	14.8	97.8

**Table 6:** Experiments Repeated on the Encoded Target Dataset (mean ± std of 5 runs).

Seed	mAP@0.5	mAP@0.5:0.95	Precision	Recall	FPS
0	95.5	68.0	95.8	88.5	93
1	95.2	67.6	95.4	88.1	93.5
2	95.7	68.4	96.0	88.9	92.1
3	95.3	67.8	95.6	88.3	91.7
4	95.6	68.2	95.9	88.7	92.0
mean ± std	95.46 ± 0.21	68.00 ± 0.32	95.74 ± 0.24	88.50 ± 0.32	92.46 ± 0.76

## Data Availability

All data generated or analyzed in this study are included in this published article.

## Abbreviations

The following abbreviations are used in this manuscript:

YOLO	You Only Look Once
SSD	Single Shot MultiBox Detector
MSCN	Multi-Scale Context Fusion Network
LAM	Lighting–Occlusion Attention Mechanism
HPP	Hybrid Pooling Pyramid
DPFP	Dual-Path Fusion Pooling
FPN	Feature Pyramid Network
RPN	Region Proposal Network
UAV	Unmanned Aerial Vehicle
NMS	Non-Maximum Suppression
MLGA	Multi-Scale Lightweight Group Attention
SiLU	Sigmoid Linear Unit
C2PSA	Cross-Channel Pyramid Self-Attention
mAP	mean Average Precision
AP	Average Precision
P	Precision
R	Recall rate
